# The Effect of Academic Procrastination on Life Satisfaction Among Nursing and Midwifery Students: The Serial Mediation Role of Academic Self-Efficacy and Self-Control

**DOI:** 10.3390/bs15111434

**Published:** 2025-10-22

**Authors:** Sevda Demir, Hilal Kuşcu Karatepe

**Affiliations:** Department of Health Management, Faculty of Health Sciences, Karacaoğlan Campus, Osmaniye Korkut Ata University, Osmaniye 80000, Türkiye; hkuscukaratepe@osmaniye.edu.tr

**Keywords:** academic procrastination, life satisfaction, academic self-efficacy, self-control, nursing students, midwifery students, serial mediation

## Abstract

This study examines the effect of academic procrastination on life satisfaction among nursing and midwifery students and explores the serial mediating role of academic self-efficacy and self-control in this relationship. Academic procrastination is a common issue among students and is negatively associated with low life satisfaction. Understanding the mechanisms underlying this relationship is important for developing effective interventions. The study included 467 nursing and midwifery students from a state university. Data were analyzed using SPSS and AMOS to test the serial mediation model. Academic procrastination was found to have significant negative effects on academic self-efficacy (β = −0.521, *p* < 0.001), self-control (β = −0.556, *p* < 0.001), and life satisfaction (β = −0.268, *p* < 0.001). Both academic self-efficacy (β = 0.242, *p* < 0.001) and self-control (β = 0.317, *p* < 0.001) significantly predicted life satisfaction. The total indirect effect of academic procrastination on life satisfaction was significant, with academic self-efficacy and self-control acting as significant serial mediators (β = −0.349, 95% CI [−0.452, −0.235]). Academic procrastination reduces life satisfaction among nursing and midwifery students, but this negative effect can be mitigated through enhanced academic self-efficacy and self-control.

## 1. Introduction

Nurses and midwives are a vulnerable group in terms of physical and psychological health (Melnyk et al., 2018; Schooley et al., 2016). Increasing demands on a global scale, problems in working conditions, difficulties in recruitment and retention of nurses and midwives have further deepened the negativities related to the profession (Donaghy et al., 2022; Yu et al., 2022; WHO, 2020). A systematic review by Priano et al. (2018) reported that nurses experience higher rates of depression, burnout, and exhibit less healthy lifestyle behaviors and work–life balance.

Previous literature has also described the nursing and midwifery education process as an intense, stressful and emotionally challenging experience (Bodys-Cupak et al., 2019a; Chen & Hung, 2014; García-Izquierdo et al., 2018; Gibbons, 2010; Onan et al., 2019; Ríos-Risquez et al., 2018). This experience is due to various factors such as assuming responsibility for the health and life of others, heavy academic load, exam pressure, negative clinical experiences, and lack of free time (Crombie et al., 2013; García-Izquierdo et al., 2018; Gibbons, 2010). Especially in practical courses, students face stressful situations such as patient care, performing medical procedures, communicating with relatives and collaborating with multidisciplinary teams (Drew et al., 2016; Kaneko & Momino, 2015; Dev Bhurtun et al., 2021; Bodys-Cupak et al., 2019a). Bodys-Cupak et al. (2019b) emphasized that the adaptation to daily life and life satisfaction of students with high levels of anxiety are negatively affected.

**Figure 1:**
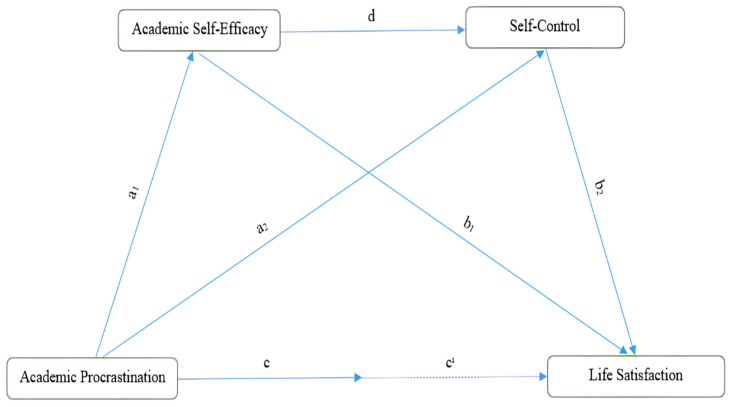
Research model.

The theoretical basis of this research is based on Social Cognitive Theory (Bandura, 1986). Social Cognitive Theory argues that an individual’s behaviors are shaped not only by external rewards or punishments, but also by the belief in one’s own competence and the ability to direct one’s behaviors accordingly (Schunk & DiBenedetto, 2020). Moreover, it argues that individuals’ behaviors are shaped through the interaction of cognitive, affective and environmental factors. In particular, it emphasizes that individual resources such as self-efficacy and self-control play a decisive role in an individual’s learning processes, motivation and psychological well-being (Bandura, 1997; Zimmerman, 2000). In this case, it is important to know and develop the importance of resources. Otherwise, according to the self-control resources theory and the self-control power model, a person’s self-control capacity is finite like the “power” of his/her muscles. According to the ego depletion theory, individuals’ psychological resources are limited and when faced with stressors, individuals mobilize their internal resources to resist. When internal resources are depleted, mobilization is hindered, resulting in significant health problems (Fredrickson, 2001). Based on this, the research model (Figure 1) investigated the mitigating roles of academic self-efficacy and self-control in the negative effect of academic procrastination on life satisfaction.

## 2. Theoretical Framework

Academic procrastination is a maladaptive behavior based on delaying or not performing a pre-planned task without any clear justification (Johansson et al., 2023). According to the Temporal Decision-Making Model (TDM), this behavior occurs when the short-term difficulties associated with performing the task outweigh the possible long-term benefits (S. Zhang & Feng, 2020). According to this model, students tend to engage in activities that provide instant gratification (e.g., relaxation, social media, entertainment) rather than long-term academic goals (Kim & Seo, 2015; Afzal & Jami, 2018).

At the university level, it has been reported that more than 70% of students regularly procrastinate academic tasks and that this behavior is common in the learning process (Schraw et al., 2007). It has been emphasized that academic procrastination is also common, especially among students studying in programs with intense professional responsibilities such as nursing, and can lead to damaging consequences such as low academic achievement, high stress, and especially decreased life satisfaction (Custer, 2018; Flett et al., 2016; Huang et al., 2023; Kandemir, 2014; Martinčeková & Enright, 2020; Maryam et al., 2016; Savithri, 2014; Yang et al., 2022; Yao et al., 2021; Y. Zhang et al., 2018). In studies conducted in different samples, individuals’ low levels of academic self-efficacy (Klassen et al., 2008; Steel, 2007) and self-control capacities (Duckworth et al., 2019; Tangney et al., 2004) were associated with high academic procrastination.

Within the framework of Bandura’s (1997) social cognitive theory, self-efficacy is a cognitive belief system that is shaped by past experiences, coping strategies, physiological states, and social interactions and directly affects an individual’s behavioral motivation. Studies have shown that students with high academic self-efficacy perform their academic tasks more effectively and that these students resort to academic procrastination behavior less (Kandemir, 2014; Krispenz et al., 2019; C. Li et al., 2022; Liu et al., 2020; Putra & Soetjiningsih, 2023; Svartdal et al., 2022). Wäschle et al. (2014) emphasized that students with high self-efficacy exhibit less procrastination behavior and that students who tend to procrastinate enter into a vicious cycle in which they weaken their self-efficacy perceptions over time.

Self-control refers to a student’s capacity to regulate his/her behavior, sustain attention, and control impulses to achieve academic goals (Duckworth et al., 2019; Liu et al., 2020). Many studies have shown that there are significant negative relationships between academic self-control and procrastination (Kühnel et al., 2018; C. Li et al., 2022; Przepiórka et al., 2019). The chronicity of procrastination behaviors can lead to an increase in students’ academic burden, making them feel under time pressure and consequently lose their sense of control (Sirois & Pychyl, 2013; Steel, 2007). It has been determined that students with high self-control complete their academic tasks in a planned manner and behave more controlled under time pressure (Duckworth et al., 2016; Duckworth et al., 2019).

It has been shown that individuals with high self-control tend to develop positive coping strategies, which increases life satisfaction (Hofmann et al., 2014; J.-B. Li et al., 2016). Significant positive relationships were found between life satisfaction and self-control, especially in young adulthood (Lee et al., 2023; Liang et al., 2022; Wittmann et al., 2014). It has been suggested that the vicious cycle between self-efficacy and procrastination may also negatively affect life satisfaction (Judge & Bono, 2001; Kaur & Kaur, 2019). Various studies have also supported that self-efficacy positively affects self-control and that there is a mutual interaction between the two concepts (Ein-Gar & Steinhart, 2017; C. Li et al., 2022). In a study conducted in China, interventions targeting self-efficacy and self-control were found to be effective in reducing academic procrastination behavior (C. Li et al., 2022). Similarly, Liu et al. (2020) found that self-control fully mediated the relationship between self-efficacy and procrastination. The following research hypotheses have been developed within the scope of this study, based on the relationships presented in the literature.

**H_1_.**  *Academic procrastination has a negative effect on life satisfaction.*

**H_2_.**  *Academic procrastination has a negative effect on academic self-efficacy.*

**H_3_.**  *Academic self-efficacy has a positive effect on self-control.*

**H_4_.**  *Self-control has a positive effect on life satisfaction.*

**H_5_.**  *Academic self-efficacy and self-control play a serial (serial) mediating role in the relationship between academic procrastination and life satisfaction.*

## 3. Methods

### 3.1. Study Design

This study is a quantitative, cross-sectional and correlational survey model study conducted to examine the serial mediation role of academic self-efficacy and self-control in the effect of academic procrastination on life satisfaction. The study was conducted in accordance with the principles of the Declaration of Helsinki.

### 3.2. Study Setting and Participants

The study population consists of students enrolled in the nursing and midwifery departments at a state university in Turkey during the 2024–2025 academic year. In total, the population consisted of 803 students, of whom 346 were studying midwifery and 457 were studying nursing. The inclusion criteria for the study are as follows: (1) Being enrolled in the nursing or midwifery programme, (2) Having completed at least one semester, and (3) Volunteering to participate in the research. The exclusion criteria are incomplete or misleading completion of the questionnaire. During the data collection process, 490 students (61% of the population) were reached using a purposive sampling method. A total of 467 valid forms were included in the analysis, giving a response rate of 95.3%. Participation in the study was voluntary, and informed consent was obtained by explaining the purpose of the study to the participants. The socio-demographic characteristics of nursing and midwifery students are presented in Table 1.

Table 1 shows that 81.4% of the students were female, 92.3% were aged 18–24, 64.9% were in the nursing department, 50.3% were in their second year, and 87.4% did not have a regular work schedule. This study found no significant differences in the analyses conducted between gender, age, department, class and the regular study programme, and academic procrastination, life satisfaction, academic self-efficacy, self-control, self-discipline and impulsivity (see Supplementary Table S1; *p* > 0.05).

### 3.3. Data Collection Process

Data were collected using a face-to-face method in May–June 2025. Participants were given a questionnaire consisting of the following scales, for which validity and reliability studies had been conducted:

Academic Procrastination Scale: The Academic Procrastination Scale Short Form, developed by McCloskey (2011) and adapted for the Turkish context by Balkis and Duru (2022), was used in this study. This shortened version comprises five items, with participants rating each item on a five-point Likert scale ranging from “Disagree” (1) to “Agree” (5). The scale does not have a reverse coded expression. The Cronbach’s alpha value of the original scale was 0.87. The Cronbach’s alpha value for the scale adaptation study was 0.88. In our study, it was found to be 0.90. The total score that can be obtained from the scale ranges from a minimum of 5 to a maximum of 25. Higher scores indicate a greater tendency to postpone academic tasks, while lower scores suggest a stronger inclination to complete them on time.

Satisfaction with Life Scale: This study used the Satisfaction with Life Scale, developed by Diener et al. (1985), to determine the life satisfaction levels of the participants. Cronbach’s alpha coefficient was 0.87 (Diener et al., 1985). Dağli and Baysal (2016) adapted the scale for Turkish culture, achieving a Cronbach’s alpha coefficient of 0.88. In this study, Cronbach’s alpha was 0.88. The scale is a 5-item, 5-point Likert-type measurement tool designed to evaluate individuals’ general life satisfaction. The scale does not have a reverse coded expression. Participants respond to each item on a scale from “Strongly Disagree” (1) to “Strongly Agree” (5). The minimum total score that can be obtained from the scale is 5, and the maximum is 25. Higher scores indicate greater life satisfaction, while lower scores indicate lower satisfaction.

Academic Self-Efficacy Scale: This study used the Academic Self-Efficacy Scale, which was developed by Jerusalem and Schwarzer (2003), adapted into Turkish by (Yilmaz et al., 2007), and then used to determine the academic self-efficacy levels of the participants. The scale measures individuals’ perceptions of their ability to fulfil academic tasks, and is a 7-item, 4-point Likert-type measurement tool with one dimension. The scale does not have a reverse coded expression. Participants respond to each item on a scale from “Not at all true” (1) to “Completely true” (4). The original scale’s Cronbach’s alpha coefficient was 0.87. The Turkish form’s Cronbach’s alpha coefficient was 0.79. In this study, Cronbach’s alpha was 0.88. The minimum total score that can be obtained from the scale is 7, and the maximum is 28. High scores indicate a strong belief in one’s ability to successfully complete academic tasks, while low scores indicate a weak belief in this ability.

Brief Self-Control Scale: This study used the Brief Self-Control Scale (BSCS), which was developed by Tangney et al. (2004) and adapted into Turkish by Nebioglu et al. (2012), to measure the self-control levels of the participants. The scale was designed to evaluate individuals’ ability to regulate their emotions, thoughts, and behaviours. It consists of 13 items and two sub-dimensions: self-discipline and impulsivity. The scale contains four positive and nine negative items. The negative items were recoded prior to analysis. Each item uses a 5-point Likert scale ranging from “completely contrary” (1) to “completely appropriate” (5). Cronbach’s alpha coefficient was 0.85 (Tangney et al., 2004). In the Turkish adaptation study, it was calculated as 0.83 (Nebioglu et al., 2012). In this study, it was 0.76. The Cronbach’s Alpha values for the subscales of the scale were determined to be 0.73 for self-discipline and 0.75 for impulsivity. The total score that can be obtained from the scale ranges from a minimum of 13 to a maximum of 65. High scores indicate a high level of self-control, meaning the individual has developed the skills to maintain attention, regulate behaviour, and control impulses in order to achieve goals. Conversely, low scores indicate a lack of self-control skills and suggest that the individual is more susceptible to external influences.

### 3.4. Data Analysis

The Statistical Package for the Social Sciences (SPSS) software, version 26.0 (IBM Corp., Armonk, NY, USA), and the Analysis of Moments Structures (AMOS) software, version 24.0 (IBM Corp., Armonk, NY, USA) were used to analyse the data. The data showed a normal distribution (skewness = −0.265 to 0.389; kurtosis = −0.561 to 0.808) (Hair et al., 2013). Descriptive statistics were computed using SPSS version 26.0 to summarize continuous and categorical variables. Cronbach’s alpha coefficient was used to calculate the internal consistency of the scales, and Pearson’s correlation coefficient was used to determine the relationships between variables.

The mediation analysis was conducted based on the conceptual framework of Hayes’ (2022) Model 6; however, all analyses were performed in the AMOS version 24. The validation of the model and the assessment of the fit indices were carried out using AMOS version 24. This analysis enabled the evaluation of both direct and indirect effects, as well as the model’s overall fit indices.

The maximum likelihood estimation method was used to validate the relationships in the model. The model fit values were used as the basis for evaluating the model (2 < χ^2^/df ≤ 5; 0.05 < RMR, RMSEA ≤ 0.08; 0.90 ≤ NFI, CFI, IFI, RFI and TLI ≤ 0.95) (Lei & Wu, 2007). Socio-demographic variables such as age, gender and class level were tested as control variables in the model; however, as the inclusion of these variables did not significantly alter the model fit, they were not included in the final model. Additionally, parallel and reverse mediation models were tested, and the serial mediation model provided the best fit indices.

A 95% confidence interval was calculated for indirect effects using the Bootstrap method. Mediating effects were considered significant when confidence intervals did not include zero. Additionally, to minimize the risk of Type-I error due to testing multiple hypotheses, the significance level was adjusted using the Bonferroni correction (0.05/5 = 0.01). The relationships in the model remained statistically significant after this adjustment. The overall significance level was set at *p* < 0.05.

## 4. Results

Table 2 presents descriptive statistics for academic procrastination, life satisfaction, academic self-efficacy and self-control. In this study, variables such as age, gender, department and class level were not included in the model as covariates.

The grade point average for academic procrastination was 3.02 ± 1.05; life satisfaction was 3.01 ± 0.89; academic self-efficacy was 2.82 ± 0.70; and self-control was 3.42 ± 0.49. In the sub-dimensions of the self-control scale, self-discipline was found to be 3.44 ± 0.50 and impulsivity was found to be 3.40 ± 0.62 (see Table 2).

The results of the Pearson correlation analysis examining the relationship between academic procrastination, life satisfaction, academic self-efficacy and self-control are presented in Table 3.

A significant negative relationship was found between academic procrastination and life satisfaction, academic self-efficacy, and self-control. The respective correlation coefficients were −0.546, −0.478, and −0.553. Table 3 shows that a significant and positive relationship was found between life satisfaction and both academic self-efficacy (r = 0.485) and self-control (r = 0.506).

**Figure 2:**
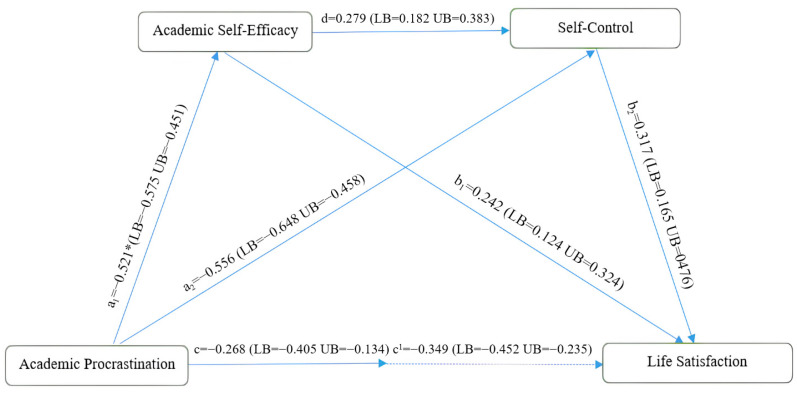
Mediation analysis results.

Figure 2 and Table 4 show the serial mediating role of academic self-efficacy and self-control in the effect of academic procrastination on life satisfaction.

In terms of direct effects, it was determined that academic procrastination had a significant negative effect on academic self-efficacy (β = −0.521, *p* < 0.001), and that the value of the explained variance (R^2^) was 0.272. In terms of self-control, it was found that academic procrastination had a negative impact (β = −0.556, *p* < 0.001), while academic self-efficacy had a positive and significant impact (β = 0.279, *p* < 0.001). The variance (R^2^) explained by academic procrastination and academic self-efficacy with regard to self-monitoring is 0.546. It was determined that academic procrastination had a negative effect (β = −0.268, *p* < 0.001), academic self-efficacy had a positive effect (β = 0.242, *p* < 0.001), and self-control had a positive effect (β = 0.317, *p* < 0.001) on life satisfaction. The explained variance value (R^2^) of academic procrastination, academic self-efficacy, and self-control on life satisfaction was determined to be 0.505.

In indirect effects, it was determined that academic procrastination behaviour has a significant and negative indirect effect on life satisfaction through academic self-efficacy and self-control (β = −0.349, 95% CI [−0.452, −0.235]). The findings reveal that academic self-efficacy and self-control play a significant mediating role in the model (Hayes, 2022; Table 4)

The validity of the proposed model was evaluated by taking into account the model fit values (Lei & Wu, 2007). The model fit values (χ^2^/df = 2.320; NFI = 0.933; RFI = 0.919; IFI = 0.961; TLI = 0.953; CFI = 0.961; RMSEA = 0.053; RMR = 0.044) indicated an acceptable level of fit (see Table 5).

## 5. Discussion

This study examined the serial mediating roles of academic self-efficacy and self-control in the possible negative effects of academic procrastination on life satisfaction among nursing and midwifery students. Using a limited set of variables and the relationships between these variables, a final model was accepted in accordance with a series of suitability criteria.

The findings show that the model, which was shaped within the framework of Bandura’s (1997) Social Cognitive Theory, is meaningful and theoretically consistent. As predicted by the model, academic procrastination negatively affects life satisfaction. But this negative effect is mitigated by academic self-efficacy and self-control. Additionally, it has been supported that academic self-efficacy positively affects self-control, and these two psychological resources have a serial effect on life satisfaction.

It was determined that the majority of the participants did not have a regular study schedule. This may indicate a high level of academic procrastination behavior. In the study, the mean score on the Academic Procrastination Scale was at a moderate-to-high level. This finding is consistent with similar studies in the literature (Brando-Garrido et al., 2020; Y. Zhang et al., 2018). Students’ life satisfaction was also found to be at a moderate level. The findings are consistent with previous research (Karataş et al., 2021; Büyükçolpan & Ozdemir, 2022). There was no significant difference in procrastination and life satisfaction between students who had a regular study schedule and those who did not. This suggests that simply having a study schedule may not be sufficient on its own, and that structured academic support programmes are necessary for effective results. Students’ self-efficacy scores were found to be moderate to high. This may suggest that, despite demanding academic programmes, students in health-related fields have positive beliefs about completing tasks and coping effectively. Previous studies have also reported moderate levels of academic self-efficacy among nursing students (İncesu & Ulupınar, 2024; Klassen et al., 2008). The level of self-control is also high, suggesting that individuals have a high capacity to regulate and sustain behaviors aimed at achieving their goals.

One of the original contributions of this study is that it reveals the serial influence of individual psychological resources by modeling the chain of academic self-efficacy → self-control → life satisfaction. The findings indicate that procrastination behavior may negatively affect life satisfaction; however, this effect can be buffered through self-efficacy and self-control, which are fundamental psychological resources of the individual (Steel, 2007; Klassen et al., 2008; Tangney et al., 2004). The findings of this study suggest that interventions aimed at enhancing academic self-efficacy and self-control should be developed to improve the life satisfaction of future healthcare professionals. Strengthening these skills among midwifery and nursing students may, in the long term, contribute to workforce sustainability, increase efficiency in the healthcare system, and enhance the quality of patient care. In particular, training programs focused on time management, goal setting, and attention control may help reduce academic procrastination.

## 6. Conclusions

The results of this study demonstrated that the negative effect of academic procrastination on life satisfaction among nursing and midwifery students can be mitigated through academic self-efficacy and self-control. The findings suggest that in designing supportive interventions, particularly for health sciences students, self-efficacy and self-control skills should be addressed together. Students’ self-efficacy can be enhanced through goal setting, receiving positive feedback related to academic achievements, and developing self-assessment skills. Self-control skills, on the other hand, can be fostered by promoting structured study habits, teaching strategies for managing attention and motivation, and supporting time management abilities.

These findings suggest that counselling and psycho-educational programmes that support self-efficacy and self-control skills may reduce procrastination behaviour in students. It is also thought that short training modules focusing on developing time management, academic self-regulation, and self-motivation skills could boost students’ life satisfaction.

In this framework, psycho-educational programs are considered to be effective in both enhancing academic achievement and supporting life satisfaction. Educational modules that focus specifically on time management, intrinsic motivation, and self-regulation skills are thought to make significant contributions to reducing academic procrastination behaviors and increasing students’ levels of self-awareness.

## 7. Limitations

This study has several limitations. First, the sample is limited to nursing and midwifery students from a single public university in Turkey, which restricts the generalizability of the findings. Second, the data were collected cross-sectionally during the May-June 2025 period. This cross-sectional design limits the ability to test causal relationships among variables. Third, all data are based on participants’ self-reports. The social desirability bias inherent in self-report measures may affect the accuracy of the responses. Fourthly, potential influencing factors such as sleep patterns, mood, or academic workload have not been included in the model. Finally, the study focused solely on individual psychological variables (academic self-efficacy and self-control). External factors such as social support, family influence, or academic environment were not included in the model.

Taking these limitations into consideration, it is recommended that longitudinal and multicenter studies be conducted. Furthermore, it is important to plan short-term evaluations to assess the effectiveness of future psychoeducational modules within the same program. Such follow-up studies may contribute to assessing the sustainability of interventions and students’ long-term academic adjustment processes.

## Figures and Tables

**Table 1:** Socio-demographic characteristics of the participants.

Variables	*N*		%
Gender	Female	380	81.4
	Male	87	18.6
Age	18–24 years	431	92.3
	25–31 years	21	4.5
	32–38 years	15	3.2
Department	Nursing	303	64.9
	Midwifery	164	35.1
Year of study	1st year	74	15.8
	2nd year	235	50.3
	3rd year	141	30.2
	4th year	17	3.6
Do you have a regular study schedule?	Yes	59	12.6
	No	408	87.4

**Table 2:** Descriptive statistics.

Variables	Min–Max	Total Average Score	Min–Max	$\bar{x}$ ± *ss*	Skewness	Kurtosis	Cronbach Alpha
Academic procrastination	5–25	15.11 ± 5.25	1–5	3.02 ± 1.05	0.039	−0.561	0.90
Life satisfaction	5–25	15.12 ± 4.48	1–5	3.01 ± 0.89	0.010	0.075	0.88
Academic self-efficacy	7–28	19.75 ± 4.93	1–4	2.82 ± 0.70	−0.265	−0.211	0.88
Self-control	13–65	44.29 ± 6.80	1–5	3.42 ± 0.49	0.389	0.461	0.76
Self-discipline	5–25	17.23 ± 2.54	1–5	3.44 ± 0.50	0.076	0.808	0.73
Impulsivity	8–40	27.06 ± 5.25	1–5	3.40 ± 0.62	0.218	0.190	0.75

**Table 3:** Pearson correlation analysis results.

Variables		Life Satisfaction		Academic Self-Efficacy		Self-Control	
Academic procrastination	r	−0.546 **		−0.478 **		−0.553 **	
%95 CI (LB UB)		−0.617	−0.479	−0.550	−0.406	−0.623	−0.487
Life satisfaction	r			0.485 **		0.506 **	
%95 CI (LB UB)				0.410	0.557	0.421	0.525
Academic self-efficacy	r					0.454 **	
%95 CI (LB UB)						0.370	0.527

** *p* < 0.001; CI: Confidence Interval; LB: Low Bound; UB: Upper Bound.

**Table 4:** Mediation analysis results.

Variables	SC	USC	SE	T	*p*	R^2^	%95 CI	
							LL	UL
Direct effect								
AP → ASE	−0.521	−0.319	0.034	−9.363	<0.001	0.272	−0.575	−0.451
AP → SC	−0.556	−0.256	0.029	−8.923	<0.001	0.548	−0.648	−0.458
ASE → SC	0.279	0.210	0.046	4.565	<0.001		0.182	0.383
AP → LS	−0.268	−0.193	0.052	−3.676	<0.001	0.505	−0.405	−0.134
ASE → LS	0.242	0.285	0.070	4.049	<0.001		0.124	0.324
SC → LS	0.317	0.285	0.143	3.481	<0.001		0.165	0.476
Indirect effect								
AP → ASE → SC → LS	−0.349	−0.251	0.051	−4.921	<0.001		−0.452	−0.235
Total effect								
AP → LS	−0.617	−0.444	0.041	−9.048	<0.001		−0.669	−0.546

*p* < 0.001; SC: Standardised Coefficients; USC: Unstandardised Coefficients; SE: Standard Error; T: Test Value; CI: Confidence Interval; LL: Lower Limit; UL: Upper Limit; AP: Academic Procrastination; LS: Life Satisfaction; ASE: Academic Self-Efficacy; SC: Self-Control. Arrows indicate the hypothesized direction of direct and indirect effects between variables.

**Table 5:** Model goodness-of-fit indices (*N* = 467).

	χ^2^/df	NFI	RFI	IFI	TLI	CFI	RMSEA	RMR
Good fit	≤3	≥0.95	≥0.95	≥0.95	≥0.95	≥0.95	≤0.05	≤0.05
Acceptable fit	≤5	≥0.90	≥0.90	≥0.90	≥0.90	≥0.90	≤0.08	≤0.08
AP → ASE → LS	2.112	0.947	0.936	0.971	0.965	0.971	0.049	0.046
AP → SC → LS	2.542	0.960	0.946	0.975	0.967	0.975	0.058	0.041
AP → ASE → SC → LS	2.320	0.933	0.919	0.961	0.953	0.961	0.053	0.044

χ^2^/df: chi-square/degrees of freedom; NFI: Normed Fit Index; RFI: Reporting Fit Index; IFI: Incremental Fit Index; TLI: Tucker–Lewis index; CFI: Comparative Fit Index; RMSEA: Root Mean Square Error of Approximation; RMR: Root Mean Square Residual.

## Data Availability

The datasets generated and/or analyzed during the current study are not publicly available due to privacy and confidentiality agreements with participants, but they are available from the corresponding author upon reasonable request.

## Supplementary Materials

The following supporting information can be downloaded at https://www.mdpi.com/article/10.3390/bs15111434/s1, Table S1: Evaluation of Academic Procrastination, Life Satisfaction, Academic Self-Efficacy, Self-Control, Self-Discipline, and Impulsivity in Relation to Demographic Variables.
