# Supplementary material for: The Effect of Academic Procrastination on Life Satisfaction Among Nursing and Midwifery Students: The Serial Mediation Role of Academic Self-Efficacy and Self-Control
Source: Behav Sci (Basel). 2025 Oct 22;15(11):1434. doi: 10.3390/bs15111434 (PMC12649465; doi:10.3390/bs15111434)
Supplement: Supplementary file 1 [file behavsci-15-01434-s001.zip › behavsci-3820509-supplementary.pdf]

Supplementary Table S1. Evaluation of Academic Procrastination, Life Satisfaction, Academic Self-Efficacy, Self-Control, Self-Discipline, and Impulsivity in Relation to Demographic Variables

Variables	N	Academic Procrastination	Life Satisfaction	Academic Self-Efficacy	Self-Control	Self-Discipline	Impulsivity
Gender							
Female	380	14.99±5.12	15.05±4.32	19.67±4.87	44.47±6.54	17.22±2.49	27.25±5.02
Male	87	15.62±5.82	15.41±5.12	20.11±5.21	43.49±7.84	17.26±2.77	26.22±6.14
Test value		T:-1.001	T:-0.668	T:-0.752	T:1.214	T:-0.126	T:1.635
P value		P:0.317	P:0.505	P:0.453	P:0.225	P:0.900	P:0.103
Age							
18-24 years	431	15.24±5.23	15.11±4.43	19.72±4.87	44.18±6.86	17.20±2.53	29.98±5.34
25-31 years	21	13.33±3.13	15.90±5.05	20.71±5.44	44.00±5.22	17.19±2.96	26.80±3.20
32-38 years	15	13.86±7.57	14.33±5.12	19.26±6.15	47.73±6.43	18.06±2.15	29.66±4.59
Test value		F:1.757	F:0.552	F:0.476	F:1.995	F:0.830	F:1.921
P value		P:0.174	P:0.576	P:0.622	P:0.137	P:0.437	P:0.148
Department							
Nursing	303	15.40±5.44	14.92±4.64	19.98±5.07	43.95±6.94	17.09±2.55	26.86±5.44
Midwifery	164	14.56±4.86	15.50±4.14	19.31±4.66	44.92±6.52	17.49±2.52	27.42±4.90
Test value		T:1.648	T:-1.334	T:1.415	T:-1.467	T:-1.629	T:-1.109
P value		P:0.102	P:0.183	P:0.158	P:0.143	P:0.104	P:0.268
Year of study							
1st year	74	15.23±5.25	14.38±4.89	19.78±4.13	44.68±6.78	17.43±2.75	27.25±5.31
2nd year	235	14.79±5.39	15.56±4.69	19.28±5.29	44.62±7.29	17.30±2.73	27.32±5.49
3rd year	141	15.71±5.09	14.83±3.80	20.54±4.61	43.80±5.92	17.09±2.03	26.70±4.75
4th year	17	14.41±4.56	14.29±3.80	20.17±4.88	41.52±5.72	16.29±2.17	25.23±5.20
Test value		F:0.989	F:1.870	F:1.909	F:1.448	F:1.121	F:1.117
P value		P:0.398	P:0.134	P:0.127	P:0.228	P:0.340	P:0.342
Do you have a regular study schedule?							
Yes	59	15.71±6.25	14.32±5.59	19.74±5.75	44.16±7.30	17.28±3.05	26.88±5.29
No	408	15.02±5.10	15.24±4.29	19.75±4.81	44.31±6.74	17.22±2.46	27.08±5.26
Test value		T:0.938	T:-1.473	T:-0.017	T:-0.149	T:0.176	T:-0.279
P value		P:0.349	P:0.142	P:0.987	P:0.881	P:0.860	P:0.781
